# Tyrosine Sulfation as a Protein Post-Translational Modification

**DOI:** 10.3390/molecules20022138

**Published:** 2015-01-28

**Authors:** Yuh-Shyong Yang, Chen-Chu Wang, Bo-Han Chen, You-Hua Hou, Kuo-Sheng Hung, Yi-Chih Mao

**Affiliations:** 1 Department of Biological Science and Technology, National Chiao Tung University, 75 Po-Ai Street, Hsinchu 30068, Taiwan; E-Mails: no13123.bt99g@nctu.edu.tw (C.-C.W.); J730703@yahoo.com.tw (B.-H.C.); E01252001@gmail.com (Y.-H.H.); mugokimo@gmail.com (Y.-C.M.); 2 Department of Neurosurgery, Center of Excellence for Clinical Trial and Research, Taipei Medical University-Wan Fang Medical Center, Taipei 11696, Taiwan

**Keywords:** sulfate, organic sulfate, post-translational modification (PTM), protein tyrosine sulfation (PTS), tyrosylprotein sulfotransferase (TPST), 3'-phosphoadenosine 5'-phosphosulfate (PAPS)

## Abstract

Integration of inorganic sulfate into biological molecules plays an important role in biological systems and is directly involved in the instigation of diseases. Protein tyrosine sulfation (PTS) is a common post-translational modification that was first reported in the literature fifty years ago. However, the significance of PTS under physiological conditions and its link to diseases have just begun to be appreciated in recent years. PTS is catalyzed by tyrosylprotein sulfotransferase (TPST) through transfer of an activated sulfate from 3'-phosphoadenosine-5'-phosphosulfate to tyrosine in a variety of proteins and peptides. Currently, only a small fraction of sulfated proteins is known and the understanding of the biological sulfation mechanisms is still in progress. In this review, we give an introductory and selective brief review of PTS and then summarize the basic biochemical information including the activity and the preparation of TPST, methods for the determination of PTS, and kinetics and reaction mechanism of TPST. This information is fundamental for the further exploration of the function of PTS that induces protein-protein interactions and the subsequent biochemical and physiological reactions.

## 1. Introduction: From Inorganic Sulfate to Biologically Active Sulfation

**Figure 1:**
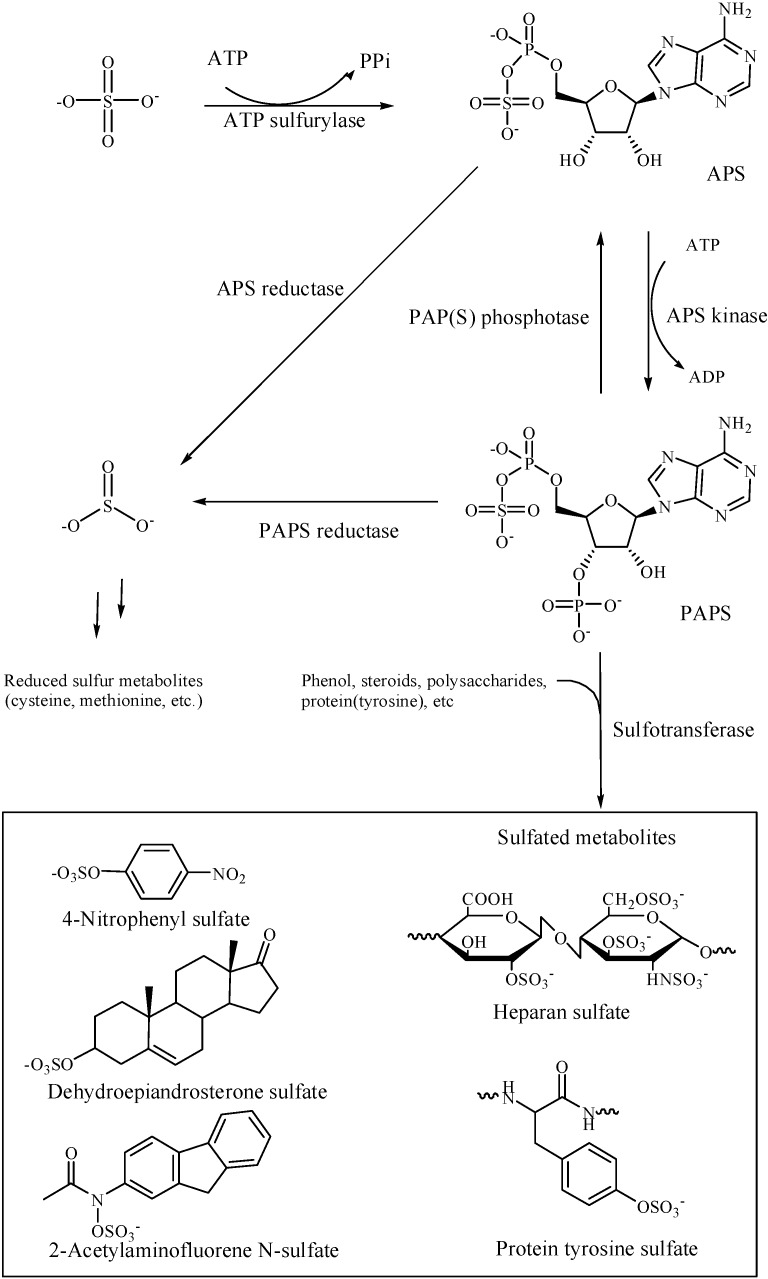
Activation of inorganic sulfate and its assimilation in biological systems. The inorganic sulfate is activated in the forms of adenosine-5'-phosphosulfate (APS) and 3'-phospho-adenosine-5'-phosphosulfate (PAPS) by ATP sulfurylase and APS kinase, respectively. Sulfotransferases are known to be responsible for the transfer of activated sulfate to a variety of biological molecules. Alternately, activation of sulfate serves as a route for the reduction and assimilation of the inorganic sulfate into important amino acids.

Organic sulfates are commonly found in biological systems. The element sulfur is available to organisms mainly in the form of inorganic sulfate, which requires metabolic activation to be further utilized by organisms as depicted in Figure 1. The activated sulfate compound is a phospho-sulfate anhydride in adenosine-5'-phosphosulfate (APS) and 3'-phosphoadenosine-5'-phosphosulfate (PAPS). Families of sulfotransferases are known to be responsible for the transfer of activated sulfate from PAPS to a variety of biological molecules such as hormones, neurotransmitters, carbohydrates, and tyrosine on proteins (Figure 1). Alternately, APS and PAPS are also used for the synthesis of reduced sulfur metabolites such as methionine and cysteine that are constituents of many proteins.

### 1.1. Sulfate Activation for Biological Assimilation

Activation of inorganic sulfate through the synthesis of APS and PAPS is a crucial step for the biological assimilation and utilization of sulfate molecules. As shown in Figure 1, PAPS is synthesized by ATP sulfurylase and APS kinase in two steps. Inorganic sulfate reacts with ATP to form APS and pyrophosphate followed by the reaction of APS with ATP to form PAPS and ADP. ATP sulfurylase and APS kinase are found on separate polypeptide chains in bacteria, fungi, yeast, and plants but are fused together into a bifunctional PAPS synthase. PAPS serves as the main activated sulfate group donor form for sulfotransferase reactions [1]. Activation of sulfate also serves as a route for the reduction and assimilation of inorganic sulfate into important amino acids (Figure 1). Both APS and PAPS are used in the sulfate-reducing pathway. APS is preferred in plants, algae, and most bacteria, whereas PAPS is used by fungi and some bacteria, including most cyanobacteria [2].

Sulfonation instead of sulfation is also used in the literature as the terminology to describe the same process [1]. In this review, we use sulfation to describe the process of the SO^3−^ group transfer from PAPS to a biomolecular receptor. This is because sulfation is the first and more commonly used form in the literature. It is also because sulfation describes more straightforwardly the whole process, which begins from the activation of inorganic sulfate and results in an organic sulfate.

### 1.2. Sulfotransferase: Enzymes that Catalyze Biological Sulfation

Sulfotransferases can be classified into three major families, cytosolic sulfotransferase, carbohydrate sulfotransferase and tyrosylprotein sulfotransferase (TPST), according to their distinct substrate selection and the location of the enzymes. Cytosolic sulfotransferases are soluble and carbohydrate sulfotransferase and TPST are membrane-associated proteins.

Cytosolic sulfotransferases catalyze sulfation of a wide variety of endogenous compounds, including hormones and neurotransmitters as well as drugs and xenobiotics [3]. The cytosolic sulfotransferases have very broad substrate specificities. They mainly select substrates with two major functional groups, the phenols and alcohols, and accordingly are categorized into two groups, the aryl sulfotransferases and the hydroxysteroid sulfotransferases. A more detailed classification of cytosolic sulfotransferases has been proposed according to their cDNA sequence [4].

The membrane-associated sulfotransferases are located in the *trans*-Golgi complex and can be classified into two classes: carbohydrate sulfotransferases and TPST. The carbohydrate sulfotransferases catalyzed the sulfation of glycolipid, glycoproteins, and proteoglycans, which mediate cell communication [5]. It has been known that sulfated carbohydrates inhibit infection by pathogens [6]. TPST has been the least studied among the three major families of sulfotransferases and will be the focus of this review.

### 1.3. Biological Function of Protein Tyrosine Sulfation (PTS)

**Figure 2:**
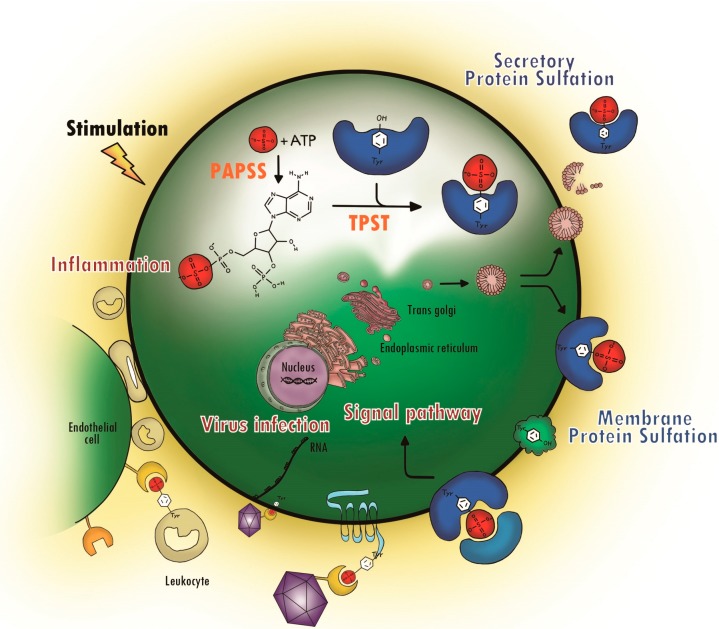
Protein tyrosine sulfation (PTS) and its biological path. The drawing depicts biochemical processes of sulfate in cell, from the activation of inorganic sulfate, its integration into protein and its effects on protein-protein interaction that induces physiological and pathogenic responses.

Protein tyrosine sulfation (PTS) is a common post-translational modification that was first reported in the literature fifty years ago [7] regarding the discovery of sulfated fibrinogen. However, the significance of PTS under physiological conditions and its link to diseases have just begun to be appreciated in recent years. Figure 2 depicts the biochemical processes of sulfate in the cell, from the activation of inorganic sulfate and its integration into proteins to its effects on protein-protein interactions that induce physiological and pathogenic responses. TPST and tyrosine-sulfated protein are involved in many biological processes, including hemostasis, leukocyte rolling on endothelial cells, visual functions, viral entry into cells and some ligand binding to receptors [8,9,10,11,12,13,14,15]. Many of these tyrosine-sulfated proteins participate in protein-protein interactions driven by recognition, at least in part, of the sulfate group [16]. So far, only secreted and transmembrane proteins are reported to have PTS. Nuclear and cytoplasmic proteins have not been reported to have PTS modification [12,17,18].

## 2. TPST Sources and Its Preparation: Enzyme Preparations, Sequences and Properties

### 2.1. Distribution of TPST Activity in Organism

TPST activities were found in a variety of cells and tissues. PTS was initially demonstrated in cell lysate from rat pheochromocytoma PC12 cells [19]. After that, TPST activities have been characterized in numerous cell lines and tissues, including mammalian tissues such as bovine adrenal medulla [20] and heart [21], rat brain [22], liver [23,24,25], gastric mucosa [26], and submandibular salivary glands [27,28], human liver [29,30], gastric mucosa [31], saliva [32], and platelets [33]. Human TPST mRNA expression level was analyzed in various human tissues (adrenal gland, bone marrow, brain, colon, heart, kidney, liver, lung, pancreas, peripheral leukocytes, placenta, prostate, salivary gland, skeletal muscle, small intestine, spinal cord, spleen, stomach, testis, thymus, thyroid gland, trachea, and uterus) by real-time reverse transcription PCR [34]. These results provide information for studies concerning the human TPST genes in various tissues. TPST activity was found to be highest in fractions enriched in Golgi membrane vesicles [19]. It has also been shown that the orientation of the catalytic site of the enzyme faces the Golgi lumen and is tightly associated with the Golgi membrane, possibly as an integral membrane protein. Other biochemical evidence has confirmed that the enzyme is an integral membrane protein, located in the trans-Golgi network, with a luminally oriented active site [19,35]. Therefore, many studies have employed mostly Golgi-enriched membrane fractions by target species tissue as the source of TPST1 and TPST2 activities [36].

TPST activity was detected in microsomal fractions of various plant species including *Asparagus officinalis* L., *Oryza sativa* L., *Daucus carota* L., *Lycopersicon esculentum* Mill., *Nicotiana tabacum* L. [37] and *Arabidopsis* [38]. Although plant and mammalian TPST share similar enzyme activity, sequence similarity searches have not identified any homology in the TPST sequences as described in Section 2.3.

### 2.2. Preparation and Purification of TPST

It is critical to obtain purified TPST for any detailed study of the biochemistry of PTS. Although TPST activity has been observed in a wide range of organisms and tissues, only limited reports mention the purification of TPST to a homogeneous form. Likely reasons for the difficulty of purifying TPST may be because it is a membrane protein and is present in restricted amounts in the cell. Table 1 lists TPST that has been purified and reported in the literatures.

Several types of affinity chromatography were developed to facilitate TPST purification. A peptide affinity column was employed for TPST purification in the presence of PAP nucleotide. The 11-aa PKV^Y+^ substrate peptide, KAALEKDYEEV, corresponding to a putative tyrosine sulfation site of α-tubulin was used as an affinity column to purify TPST from bovine adrenal medulla [39,40,41]. Similarly, a 15-aa acidic motif peptide of plant peptide containing sulfated peptide 1 (PSY1) precursor polypeptide (pPSY1) was immobilized onto Sepharose via a C-terminal Lys residue and used as an affinity matrix for TPST purification from *Arabidopsis* [38]. Combination of a Cibacron blue F3GA affinity column and anti-TPST antibody column chromatography was used to purify TPST from the Golgi membranes of rat submandibular salivary glands [28]. Similar immunoaffinity chromatography was used to purify TPST from human saliva [32].

**Table 1:** Purification of tyrosylprotein sulfotransferase (TPST) ^a^.

Species	Source	Treatment	Reference
Bovine	Adrenal medulla	peptide affinity column	[39]
Rat	Liver	anti-TPST antibody column	[25]
Rat	Submandibular salivary glands	anti-TPST antibody column	[28,42]
Mouse TPST2	HEK293-T cell	affinity column	[43]
Human	Saliva	anti-TPST antibody column	[32]
Human TPST1, TPST2	HEK293-T cell	affinity column	[43,44,45]
Human TPST2	CHO Cell	peptide affinity column	[46]
Human TPST2	*E. coli* BL21-CodonPlus(DE3)-RIL strain	affinity column	[47]
Human TPST1, TPST2	SF9 insect cell	affinity column	[48]
*Drosophila melanogaster*	*E. coli* BL21(DE3)pLysS Competent Cell	affinity column	[49]
*Arabidopsis*	Yeast	peptide affinity column	[38,50]
Zebrafish TPST1	COS-7 cell	affinity column	[51]
*Caenorhabditis elegans* TPST1	HEK293-T cell	affinity column	[52]

Notes: ^a^ This Table includes a list of sources from where TPST were purified. Same TPST species can be obtained from different sources with DNA recombinant technology.

At the current stage, a method to separate TPST1 and TPST2 has not been reported. Two TPST species (TPST1 and TPST2) were likely to be co-purified from the procedures described above when the enzyme sources were not genetically modified. Preparation of recombinant TPST not only significantly increased the abundance of the target enzyme but also ensured the preparation of the desired type of the enzyme. Purification procedures were developed to purify recombinant TPST1 and TPST2, respectively, by various affinity column chromatographies [43,45]. Recombinant TPSTs had been expressed in many cell, such as human embryonic kidney 293 cell (HEK293-T cell), Chinese hamster ovary cell (CHO Cell), *Spodoptera frugiperda* cell (SF9 insect cell), African green monkey kidney cell (COS-7 cell), Yeast, *E. coli* BL21(DE3)pLysS Competent Cell, *E. coli* BL21-CodonPlus(DE3)-RIL strain [38,43,44,45,46,47,48,49,50,51,52]. Purification of recombinant TPSTs from human, mouse, Zebrafish, *Arabidopsis*, *Drosophila melanogaster*, *Caenorhabditis elegans* have been reported [25,28,32,38,39,43,44,45,46,47,48,49,50,51,52]. A more detailed description regarding cloning and expression of TPST is given in the next section.

### 2.3. TPST Cloning, Sequence, and Structural Analysis

Two different TPSTs (TPST1 and TPST2) from humans and mice, respectively, were first identified through molecular cloning [43,45,46]. Similar studies also revealed TPST in other vertebrates, such as zebrafish [51], and invertebrates including *Caenorhabditis elegans* [52] and *Drosophila melanogaster* [49]. While most species have two TPST genes, it is interesting to find that *Drosophila melanogaster* lacks a second TPST gene [18]. In addition, plant TPST genes were found to be unique in their sequence as compared to those of vertebrate and invertebrate species [53]. It is also noted that an enzyme purified from anaerobic *Eubacterium* A-44 in the human intestine shows TPST activity. However, its DNA sequence reveals little homology with other TPST cDNA and this enzyme does not use PAPS (Figure 1) as a substrate [54]. The following TPST cDNAs from a variety of species can be found in the NCBI gene data bank: *Bos taurus* (cattle), *Gallus gallus* (chicken), *Sus scrofa* (pig), *Canis lupus* familiaris (dog), *Xenopus* (Silurana) *tropicalis* (Western clawed frog), *Cricetulus griseus* (Chinese hamster), *Macaca mulatta* (Rhesus monkey), *Pan troglodytes* (chimpanzee), *Callorhinchus milii* (elephant shark), *Equus caballus* (horse), *Acyrthosiphon pisum* (pea aphid), and *Cucumis melo* (muskmelon). It is interesting that no similar TPST gene has been found in yeast and other fungi.

TPST gene sequence analysis may reveal many important enzyme properties [55]. For example, human TPST gene sequence analysis indicates that human TPST1 and TPST2 have 370 and 377 amino acids, respectively, with predicted molecular masses of 42.2 and 41.9 kDa, respectively [43,45]. Human TPST1 and TPST2 share 63% amino acid sequence identity, which is even higher (77%) over the predicted catalytic domain residues 69–352. The region around the putative transmembrane helix (residues 26–68) has remarkably low in sequence identity between the two isoforms. Interestingly, substantially higher sequence identity (>90%) is found between each human enzyme and its corresponding enzyme in other mammalian species. Consistent with biochemical data described in Section 2.1, both TSPT1 and TPST2 are predicted to have type II transmembrane topology with a short eight-residue N-terminal cytoplasmic domain, a 17-residue transmembrane domain and a luminal catalytic domain. Post-translational sites on TPST were also predicted, each human enzyme has two potential N-linked glycosylation sites and six conserved cysteine residues in the luminal domain, suggesting the possible formation of three disulfide bonds [18,56]. TPST enzyme properties predicted from gene sequence analysis are useful, but need to be further verified because experimental data on the direct biochemical characterization of TPST are very limited. Plant TPST is a type I transmembrane protein that shows no sequence similarity to animal TPST [38]. The crystal structure of TPST2 has been solved [47]. This information provided fundamental knowledge for the continued understanding of how TPST functions as a biocatalyst for PTS. A more detailed discussion is given in Section 4.2.

## 3. Methods for the TPST Assay and Detection of PTS

Current methods available for PTS detection are in sharp contrast to those for the detection of protein phosphorylation [57]. Protein phosphorylation has been extensively studied for decades with the emergence of a variety of appropriate biochemical tools [58,59]. However, only limited tools are available for the study of PTS. The most frequent method used for the detection of PTS is to incorporate the radioactive isotope, ^35^S, into its protein/peptide substrates. Most of the previously reported sulfated proteins were verified by radioactive labeling. The other methods developed for the determination of protein sulfation generally used the known sulfated proteins as targets. However, there is one example in the literature to show that high resolution FT MS was used to directly identify the sulfation site of a secreted peptide [60]. Specific monoclonal antibodies against sulfotyrosine facilitate the detection of PTS by their high specificity and easy incorporation with well-established immunoblotting [61]. Although, nowadays, mass spectrometry is the most powerful tool in the study of post-translational modification (PTM), its application for the analysis of sulfotyrosine remains a challenge due to the liable decomposition of the sulfate group in both positive and negative ion tandem mass spectrometry (MS/MS) experiments that may result in a false negative result [62,63]. Very recently, a fluorescence method for the determination of TPST activities and PTS was reported [48,49]. Powerful techniques such as two-dimensional gel electrophoresis (2-DE) and mass spectrometry (MS) [64] developed for PTM functional proteomics studies, especially for phosphoproteomics [59,65], have not been reported in the literature for sulfoproteomic research studies. Currently known sulfated candidates are grouped into secretory and transmembrane proteins, which are not easily compatible with the methods developed for traditional 2-DE based proteomics. Membrane proteins are characterized by hydrophobic domains, and are thus susceptible to precipitation and disturbance due to their low solubility during first dimensional isoelectric electrophoresis, precluding them to be transferred into the second dimension of 2-DE process. Methods reported for the analysis of PTS are summarized in Table 2. Detailed descriptions are given in the following sub-sections.

**Table 2:** Methods for the assay of TPST activity and the detection of PTS ^a^.

Method	Sensing Target	Sensing Principle	Advantages	Disadvantage	Reference
Radiometric	^35^S	Radioactive PAPS as ^35^SO_3_^−^ donor	Highly sensitive and direct	Discontinuous assay and tedious procedure	[25,66]
Colorimetric	Antibody	Sulfated protein recognized by anti-sulfotyrosine	High-throughput	Less sensitive and discontinuous assay	[61,67]
Fluorimetric	MU ^b^	PST and TPST coupled enzyme assay to produce fluorescent signal	Fast, continuous, real-time and without substrate limitation	Indirect	[49]
	DAP ^c^-pyrene	Tyrosine sulfation disrupt π-π stacking interactions to produce fluorescent signal	Fast, continuous, real-time and direct	Limitation in peptide sulfation; synthetic fluorescent amino acid conjugated in peptide	[48]
Mass spectrometric	Mass variation	Mass transfer (-SO_3_^−^) detected by MS	Highly sensitive and accurate	Discontinuous assay, sulfated protein unstable in mass process and instrument dependent	[36]

Notes: ^a^ This Table summarizes methods reported for the determination of TPST activity and analysis of PTS, which base on difference principle for the detection of sulfated proteins; ^b^ MU: 4-methylumbelliferone; ^c^ DAP: l-2,3-diaminopropionic acid (DAP).

### 3.1. Isotope Labeling for the Detection of PTS

Isotope labeling has been the most frequently used method for the detection of biological sulfation and for sulfotransferase assays including cytosolic sulfotransferase [68], carbohydrate sulfotransferase [69], and TPST [25]. This method uses radioactive isotope-labeled [^35^S] PAPS as the SO_3_^‒^ group donor to a variety of sulfation sites including small molecules, glycan on glycoprotein and tyrosine on peptides or proteins substrates. The ^35^S labeled sulfated peptides or proteins are usually separated from the reaction mixture following TPST catalyzed PTS for the determination of their radioactivity. The tyrosine ^35^S sulfated product can be separated from the mixture of the TPST assay by thin-layer chromatography (TLC) or polyacrylamide gel electrophoresis [66,70] and exposed to X-ray film for the detection of a sulfation reaction and the TPST activity. The positions of the sulfated product and [^35^S] PAPS were identified by overlaying the autoradiogram after the film was developed. Alternatively, the hot spots were cut and counted with scintillation counter to determine the activity of tyrosine ^35^S sulfation.

Radioactive isotope labeling of sulfate, ^35^SO_4_^2‒^, was also used directly in the medium of mammalian cell cultures to investigate PTS *in vivo* [71]. In a cell system, radioactive inorganic sulfate can be activated by PAPS synthetase (Figure 1) to form radioactive PAPS. A similar transformation was also used to produce radioactive PAPS *in vitro* [72] for the sulfotransferase assay and for the production of organic sulfates. Using radioactive isotope labeling at ^35^S and ^14^C, about 1% sulfated tyrosine residues were estimated in fly proteins [73]. Radioactive sulfate labeling of proteins can label both carbohydrates and tyrosine residues under the conditions described above. Bacterial aryl sulfatase was used to confirm TPS [74] by removing the radioactive sulfate. However, aryl sulfatase can be non-specific [75] and may catalyze hydrolysis of both carbohydrate and tyrosylprotein sulfates. Glycosidase was used to hydrolyze carbohydrate from glycoprotein [76], which specifically removes carbohydrate sulfation and leaves PTS intact. The anti-sulfotyrosine antibody described in Sub-section 3.3 can be used to confirm tyrosylprotein sulfation. Analysis of sulfated protein by MS can distinguish the sulfation site within each protein as described in the following sub-section, it would be a powerful technique to simultaneously identify different types of sulfation.

Radioactive isotope labeling ^35^S is very useful to determine a small amount of sulfated proteins. This is particularly important for the products of TPST, for most tyrosine-sulfated proteins are membrane and secretory proteins [77,78], and membrane proteins are known to be very difficult to purify and analyze [79]. A major disadvantage of using radioactive isotope labeling is its complex operation procedure and regulations.

### 3.2. PTS Determined by the Variation of Mass

MS has become one of the most powerful tools to observe various PTMs including phosphorylation, ubiquitination, glycosylation, methylation, acetylation, and sulfation through monitoring the molecular weight variation of before and after PTMs [80,81]. However, the main disadvantage for using this technique is that MS necessitates skillful and professional operation with expensive instrumentation. A previous review article has described the analysis of sulfated proteins by MS [82]. In this section, we further update the use of MS for PTS.

Sulfated tyrosine was less stable than phosphorylated tyrosine according to a study using MS/MS [80]. Detection of mass variation of before-and-after PTS may be confused with those of protein phosphorylation. Tyrosine sulfation and tyrosine phosphorylation give similar mass modifications at the same nominal mass around 80 Da (sulfation: 79.9568 Da; phosphorylation: 79.9663 Da) [83]. This small mass variation, 9.5 mDa, between tyrosine sulfation and phosphorylation can be distinguished by ultra-high accuracy mass measurements, such as Fourier transform-ion cyclotron resonance (FT-ICR) or Orbitrap [83,84,85].

Other MS-based methods have been developed to identify PTS and tyrosine phosphorylation [86,87]. The sulfate group of sulfated tyrosine displayed complete loss in positive mode MS. Following the blockage of free tyrosines through stoichiometric acetylation, the presence of sulfotyrosine is indicated by the detection of free tyrosine after MS/MS [87]. Alkaline phosphatase treatment can be used to distinguish phosphorylation and sulfation of tyrosine. The method was further modified by using Br-Tag that specifically incorporates into tyrosine residues. Following treatments with phosphatase or sulfatase, the phosphotyrosine or sulfotyrosine sites can be determined unequivocally utilizing the Br signature [86].

Multiple tyrosine sulfations within a protein sulfation site also exist and this is an important factor for protein-protein interactions and other biological functions induced through PTS. Most chemokine receptors contain several potentially sulfated tyrosine residues in their extracellular N-terminal regions, where the initial binding site for chemokine ligands is found [88]. Detection of multiple tyrosine sulfation sites has been developed for P-selectin glycoprotein ligand-1 (PSGL-1) and C-C chemokine receptor type 5 (CCR5). Sulfation is the critical step for both proteins to induce virus infection [10,89]. Multiple tyrosine sulfations in a peptide was identified by liquid secondary-ion mass spectrometry (LSIMS) and matrix-assisted laser desorption/ionization time of flight mass spectrometry (MALDI-TOF MS) [63,90]. The two sulfated tyrosines can be distinguished by comparing positive- and negative-ion spectra of the sulfated peptide due to their weak and differential stability. Phosphorylated tyrosine was stable in positive and negative ion modes of MS [91].

### 3.3. Anti-Sulfotyrosine Antibody as a Tool for the Detection of PTS

Anti-sulfotyrosine antibody has been developed and used to monitor PTS by ELISA, Western blotting, and flow cytometry [61,89,92]. The main advantage of the immunoassay for the identification of PTS in biological systems is its ability for high-throughput screening. Currently a commercially available anti-sulfotyrosine monoclonal antibody (called PSG2) was found to bind with high affinity and exquisite specificity to sulfotyrosine residues in peptides and proteins independently of sequence context [61]. There is preference for this PSG2 antibody to recognize the presence of Glu or Asp within ± two residues of the sulfotyrosine, and it is a negative predictor to Lys being at the P1' position (sYK, the presence of lysine next to the sulfated tyrosine at carboxyl terminus direction) [61]. So far, there is no indication that this PSG2 antibody recognizes phosphotyrosine peptide and the sulfotyrosine residue alone. The dissociation constants of this anti-sulfotyrosine antibody and sulfated hirudin fragment 54–65 (GDFEEIPEEYLQ), gastrin-17 (EGPWLEEEEEAYGWMDF), and cholecystokinin octapeptide 26–33 (CCK8, DYMGWMDF), respectively, were estimated to be in the micromolar range by capillary electrophoresis (CE) and surface plasmon resonance (SPR) [67].

Membrane proteins including receptors, which are vital for a variety of physiological and pathological reactions, are known to contain the major sulfation sites. A combination of anti-sulfotyrosine antibody with flow cytometry can directly monitor tyrosine sulfation of cell surface proteins. The cells were incubated with anti-sulfotyrosine antibody and followed by incubation with secondary antibodies conjugated with green-fluorescent dye. The variations in fluorescence intensity observed corresponded to the amount of tyrosine sulfation on the cell surface. This tool led to the finding that tyrosine sulfation, but not glycosylation, in the N-terminal region of PSGL-1 facilitated virus entry and replication of enterovirus 71 (EV71) in leukocytes [89].

Previous research has demonstrated that immune tools are extremely valuable to help us understand the mechanism of sulfated protein-protein interaction, which is involved in virus infection and immune response. It is interesting to compare the antibodies available against phosphorylated and sulfated proteins. Numerous antibodies are available for the specific recognition of many types of phosphorylated proteins/peptides with varied sequence preferences between antibodies [93]. For the sulfated protein/peptide, however, there is only one anti-sulfotyrosine antibody reported in the literature. Since only limited amounts of sulfated protein/peptide have been examined, it is uncertain if a single commercial PSG2 antibody recognizes all sulfated proteins in a native sample. It is very easy to see that there is an urgent need in the development of anti-sulfotyrosine antibodies against various distinct sulfated proteins.

### 3.4. Fluorescent TPST Assay

Fluorescence has been used for monitoring enzyme-catalyzed sulfation for cytosolic and membrane sulfotransferases, including amine sulfotransferase 1A3 (SULT1A3) alcohol sulfotransferase 2A1 (SULT2A1) and TPST through phenol sulfotransferase (PST) coupled-enzyme assay [49,94,95]. PST-TPST coupled-enzyme assay is a real-time fluorescent method that can conveniently be used to determine the kinetics of PTS, which is essential for understanding the interface between PTS and protein-protein interactions [49]. The fluorescence is determined by a fluometer when PST catalyzes the formation of a fluorgenic compound 4-methylumbelliferone (MU) and PAPS from MUS and PAP. PAPS is the co-substrate of TPST and the source of [SO_3_^‒^] group for the sulfation of a peptide or protein substrate. Due to the continuous production of PAPS through PAP, TPST activity observed by this fluorescence method can be free of product inhibition. In addition, this assay can be used for the preparation of sulfated protein/peptide and can be further developed to become a high-throughput method for the characterization of TPSTs and for the identification and screening of their protein substrates.

A fluorescence assay to directly monitor tyrosine sulfation in real time was recenty reported. This assay measured fluorescence released from the disruption of the π-π stacking interaction between the tyrosine and synthetic fluorescent amino acid following tyrosine sulfation [48]. This assay can be used to measure dose-dependent inhibition of PAP of PTS and to develop a high-throughput method for the identification of enzyme inhibitors through screening of compound libraries. However, the peptide substrate requires a synthetic fluorescent amino acid, DAP-pyrene (l-2,3-diaminopropionic acid, DAP, conjugated with a pyrene), nearby the tyrosine residue, which limits the substrate selection for TPST assay.

### 3.5. Other Methods for the Detection and Prediction of PTS

A protein sulfation site can be predicted by bioinformatics tools. They are simple and useful methods for preliminary screening of potential protein sulfation sites [96,97,98]. Three tools that are conveniently available through the internet are Sulfinator [96], SulfoSite [97], and PredSulSite [98]. These tools also combine other protein properties including secondary structure, physicochemical properties of amino acids, and residue sequence order information based on the data set of sulfated proteins to predict protein sulfation sites. However, due to the limitation of available experimental data for PTS, the database integrity is inadequate for developing a reliable prediction method, and it is not a guarantor for the identified proteins to be actually sulfated in cells at the present stage [66]. However, at the current stage, it is still the easiest method to roughly estimate the potential sulfation site before the tedious experimental procedure described in the previous sections has to be implemented. Furthermore, more restrictions can be enforced to improve the accuracy of the prediction of PTS as shown in Table 3, in which only secretory and membrane proteins (selected by TransMembrane prediction using Hidden Markov Models (TMHMM) and SignalP) are considered as potential TPST substrates.

**Table 3:** Prediction of tyrosine sulfation in humans ^a^.

	Total Tyrosine	Total Protein	Sulfated Tyrosines Predicted ^c^	Sulfated Protein with at Least One Hit ^c^
Whole genome	421,369	36,523	23,394	13,015
TMHMM ^b^	183,024	8923	6325	3091
SignalP ^b^	142,384	9183	5003	2812
TMHMM & SignalP ^b^	110,428	3102	1738	1302
Non-redundancy from TMHMM & SignalP ^b^	214,980	15,003	9590	4601

Notes: ^a^ The total protein sequences were downloaded from NCBI genome FTP; ^b^ The α-helical transmembrane domain and secreted proteins were calculated on TransMembrane prediction using Hidden Markov Models (TMHMM) [99] and SignalP [100] web-server, respectively. The total non-redundant α-helical transmembrane and secreted proteins were obtained from the sum of the results of TMHMM and SignalP and subtracted from the overlap of TMHMM and SignalP; ^c^ The potential sulfated proteins were evaluated by the software tool Sulfinator.

**Figure 3:**
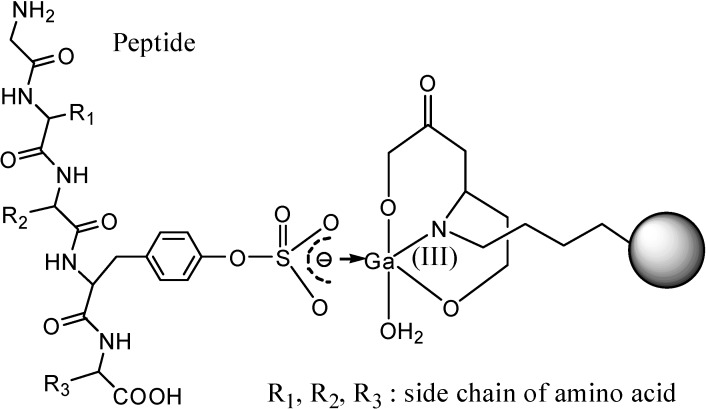
Immobilized metal ion affinity chromatography (IMAC-Ga) for the enrichment of sulfated protein. The structures of IMAC-Ga and its coordination with a sulfated tyrosine are shown. Methods for the enrichment of sulfated peptides and proteins are very useful for the detection of PTS that may be low in a cell.

Methods for the enrichment of sulfated peptides and proteins are very useful for the detection of PTS levels that may be in low in cells. Enrichment techniques are widely used prior to analysis of phosphorylation of peptides and proteins by MS [101,102,103,104]. Similar techniques for the enrichment of sulfated proteins may be developed. Immobilized metal ion affinity chromatography (IMAC-Ga) is one of the platforms for enrichment of phosphorylated proteins and has been developed to analyze PTS [105]. The structures of IMAC-Ga and its coordination with a sulfated tyrosine are shown in Figure 3. The IMAC-Ga enrichment method also enriches the phosphorylated peptides [101]. The product purified from IMAC-Ga can be further identified by mass analysis to distinguish sulfated from phosphorylated compounds. Anti-sulfotyrosine antibody has high antibody specificity for binding sulfated protein and can also be used to enrich and separate the sulfated proteins [61].

## 4. Substrates and Kinetic Properties of TPST

It is fundamental to understand the natural substrates of TPST in order to further appreciate the biological function of PTS. Currently, PTS is known to be a PTM of many secreted and membrane-bound proteins. Only about 300 such proteins have been reported in the literature and their biological functions with PTS are not well understood [57]. These sulfated proteins were found in mammals, plants, flies, and *C. elegans*. Sulfated proteins have not been reported in yeast and fungi.

This known amount of proteins may constitute only a small fraction of the total sulfated proteins, for it has been estimated that up to 1% of all tyrosine residues in the eukaryotic proteome are sulfated [73,82]. According to this amount of sulfated tyrosines measured, about 11% sulfated proteins can be roughly estimated in a fly. This estimation indicates that the currently known sulfated proteins constitute only a small fraction of the potentially total sulfated proteins. A calculation is given in Table 3 for the human proteins, and about 4600 potential sulfated proteins were predicted. There are 421,369 tyrosines among the 36,523 human proteins from the NCBI genome FTP. Sulfinator was used to predict the total amount of potential sulfated tyrosines and proteins. Total proteins obtained from NCBI were further screened for membrane and secretary proteins using TMHMM and SignalP, for those proteins are known to be the PTS candidates. Predicted results shown in Table 3 indicate that the selection of TPST substrates may not just depend on the sequence of the proteins. The transportation of proteins to the TPST location at Golgi apparatus may be a pre-requisite for PTS.

### 4.1. Biological Functions of PTS

The currently known PTS target proteins belong to the secretory, plasma membrane, and lysosomal protein classes, which reflects their intracellular localization. TPSTs catalyze the sulfation of tyrosine residues located within specific peptide sequences, which has been implicated in several crucial physiological events. The biological functions of PTS have been reported to regulate intracellular trafficking and proteolytic processing of secreted proteins. It also functions as a key modulator of extracellular protein-protein interaction by recognizing sulfated proteins, and thus mediates hormonal regulation, hemostasis, inflammation, and several infectious diseases [57,82]. In the following sections, we review examples of well-known TPST substrates that are involved in important physiological functions.

#### 4.1.1. Chemokine Receptor as PTS Sites

Chemokines are small, secreted proteins that exert many biological functions through G-protein-coupled receptors, including leukocyte trafficking, angiogenesis, angiostasis, viral infections, and host immune response to cancer [106]. Several chemokine receptors (CCR5, CXCR4, CCR2B, CX3CR1, and CXCR3) have been shown to undergo PTS [10,107,108,109,110]. PTS on CCR5 is involved in HIV-1’s entry into CD4^+^ cell. The chemokine receptor CCR5 is post-translationally modified by sulfation on its N-terminal tyrosines. Sulfated tyrosines contribute to the binding of MIP-1α, MIP-1β, and HIV-1 gp120/CD4 complexes and facilitate HIV-1 entry into cells expressing CCR5 and CD4. The N terminus of CCR5 contains four tyrosines at positions 3, 10, 14, and 15 [10], and have been modified stepwise at positions 14 or 15, followed by position 10 and finally the tyrosine residue at position 3 [44]. Mutation of the four sulfotyrosine residues in CCR5 to phenylalanine and chlorate reduces HIV infection by 50%–75%. This information suggests that inhibiting PTS of CCR5 may provide a basis for the design of therapeutic agents aimed at blocking HIV-1 cellular entry.

#### 4.1.2. PTS and Leukocyte Adhesion and Inflammatory Response

PSGL-1 is a glycoprotein found on leukocytes and endothelial cells that has a high affinity to P-selectin. The extreme amino terminus of PSGL-1 carries three potential tyrosine sulfation sites. These tyrosine sulfate esters and specific glycans on PSGL-1 are key binding determinants for P-selectin. The binding between PSGL-1 of leukocyte and P-selectin of endothelial cells is essential for leukocyte adhesion in this inflammatory response [111]. Treatment of PSGL-1 with arylsulfatase releases sulfate from tyrosine and then reduces its binding ability to P-selectin [74]. This observation is confirmed and supported by point mutagenesis of the potentially sulfated tyrosine [112]. Thus, TPST has become a therapeutic target for autoimmune diseases caused by chronic inflammation, such as rheumatoid arthritis and multiple sclerosis [113].

Some diseases, like atherosclerosis [114] and EV71 infection [115] were critically associated with PSGL-1. Sulfation on its N-terminal tyrosine residues contributes to the binding of the specific strains of EV71 through its capsid protein (VP1) [115]. EV71 strains were distinguished as EV71-PB strains (PSGL-1-binding strain) and EV71-nonPB strains (PSGL-1-non-binding strain). The infections of these EV71-PB strains resulted in encephalitis (fatal) and hand, foot, and mouth disease (HFMD) [89].

#### 4.1.3. Hemostasis and Anticoagulation

The biological function of PTS is also involved in hemostasis. PTS is crucial in the interactions between many plasma proteins such as hirudin and thrombin [116], fibronectin and fibrin [117], coagulation factor VIII, and von Willebrand factor (vWF) [11] and glycoprotein (GP) Ibα with both vWF and thrombin [118,119,120,121,122]. Moreover, platelet attachment is completed by vWF that bridges subendothelial collagen and platelet membrane protein GP Ibα. The binding between vWF and GP Ibα is dependent upon the sulfation of three tyrosines at position 276, 278 and 279. In anticoagulation, hirudin is a potent anticoagulant protein secreted in the salivary gland of the leech. The tyrosine sulfation at Tyr63 of hirudin results in a 10-fold higher affinity for thrombin than the unsulfated form, which prevents coagulation by the inhibition of thrombin [116].

#### 4.1.4. PTS on Plant Peptide Hormone

Three types of sulfated plant hormone peptides, phytosulfokine (PSK), plant peptide containing sulfated tyrosine (PSY) and root meristem growth factor (RGF) have been reviewed [123]. It is noted that PTS does not act directly on the hormone peptides, instead, the precursor proteins are the TPST substrates. Plant TPST was shown *in vitro* to modify precursors of the plant peptide hormones [38]. It is unclear if sulfation of the plant hormone peptide precursor triggered a protease cutting site to produce final plant sulfated peptide hormone. Recent reports provide more evidence to support the functions of these sulfated signaling peptides, PSY1 [124] and PSK [125].

### 4.2. Kinetic Properties and Chemical Mechanism of TPST

From 1980~2007, the Golgi membrane fraction was used as the enzyme source for many studies to analyze the TPST activity and determine its kinetic constants. In this section, we only discuss TPST kinetic constants (Table 4) determined by purified TPST (Table 1). Preparation and purification of TPST is described in Section 2.2. The V_max_ of homogenous TPSTs was hundred-fold higher than that obtained from Golgi membrane fractions. However, even the homogenous TPST purified from its original source is likely to contain the mixture of TPST1 and TPST2. The separation of TPST1 and TPST2 from cells or native source has not been reported so far in the literature. The kinetic constants of each TPST (TPST1 or TPST2) were not obtained until recombinant TPSTs fused to affinity tags were purified from prokaryotic or eukaryotic systems [36,49]. The basic kinetic constants of purified TPST from various sources were summarized in Table 4. Significant variations in kinetic constants can be observed even with the highly purified TPSTs. There are several reasons that may cause these differences shown in Table 4. First, different methods were used to monitor TPST activity. As discussed in Section 3 and listed in Table 2, current methods used for the detection of TPST activity are based on somewhat different principles and the reaction conditions may not be exactly the same. The K_m_ of protein substrates were generally determined in the micromolar range, but may vary up to 100-fold depending on the type of protein substrate and the detection method. The catalytic efficiency (*k*_cat_/K_m_) determined through real-time fluorescent assays were 10–100-fold higher than those obtained by other end-point methods, isotope labeling and MS. Kinetic studies and understanding of the catalytic mechanism of TPST are still at an infancy stage. We review three main features of tyrosine sulfation to be systematically discussed in the following sub-sections: (1) the catalytic mechanism of TPST; (2) the role of acidic acid surrounding the sulfated tyrosine residues; (3) the significance of multiple tyrosine sulfation.

**Table 4:** Kinetic constants of TPST.

Enzyme ^a^	Substrate ^b^	Detection Methods	K_m(substrate)_	K_m(PAPS)_	*k*_cat_	*k*_cat_/K_m(substrate)_^c^	V_max_	Reference
			(μM)	(μM)	(min^−1^)	(min^−1^·μM^−1^)	(nmole/min/mg)	
*r*TPST (TPST1+2)	poly-(Glu6,Ala3,Tyr1) (47 kDa)	[^35^S] PAPS	3	20	0.312 ^c^	0.104	6	[28]
*h*TPST1	CCR8, peptide	Mass spectrometry	99 ± 5	0.50 ± 0.09	0.045 ± 0.007	0.0005	1.07 ± 0.17 ^d^	[126]
	sY15CCR8, peptide		21 ± 0.1		0.02 ± 0.00	0.0010	0.47 ± 0.00 ^d^	
*h*TPST2	CCR8, peptide		120 ± 10	0.59 ± 0.10	0.50 ± 0.03	0.0042	11.9 ± 0.7 ^d^	
	sY15CCR8, peptide		23 ± 2.6		0.30 ± 0.01	0.0130	7.2 ± 0.2 ^d^	
*h*TPST (1+2) mixture (1:1)	CCR8, peptide		75 ± 4	0.54 ± 0.09	0.43 ± 0.10	0.0057		
	sY15CCR8, peptide		17 ± 0.5		0.29 ± 0.01	0.0171		
*h*TPST2	CCR8, peptide		19.3 ± 1.8	8.7 ± 0.3	0.30 ± 0.01 ^d^	0.016	7.1 ± 0.2	[36]
	sY15CCR8, peptide		3.1 ± 0.2	4.8 ± 0.8	0.075 ± 0.004 ^d^	0.026	1.8 ± 0.1	
*dm*TPST	PSGL-1, peptide	Fluorescence assay (coupled-enzyme)	53 ± 9	16 ± 4	6.2 ± 0.4	0.12	156 ± 10 ^d^	[49]
	GST fused PSGL-1 peptide		11 ± 2	3.2 ± 0.9	2.7 ± 0.1	0.25	68 ± 3 ^d^	
*h*TPST1	Fluorescent peptide, containing pyrene l-2,3-diaminopropionic acid	Fluorescence assay (fluorescence label)	1.9 ± 0.2		3.24 ± 0.12 ^e^	1.71 ^e^	77 ± 3 ^d^	[48]
*h*TPST2			1.8 ± 0.2		3.90 ± 0.12 ^e^	2.17 ^e^	93 ± 3 ^d^	

Notes: ^a^ The italics indicate the sources of TPST: *r*, *rat*; *h*, *human*; *dm*, *Drosophila melanogaster*; ^b^ Sequences of peptide substrate: (poly-(Glu6,Ala3,Tyr1), a synthetic acidic polymer contains Glu:Ala:Tyr (6:3:1); CCR8: VTDYYYPDI; sY15CCR8: VTDsYYYPDI, sY is sulfated tyrosine; PSGL-1: ATEYEYLDYDFL; GST fused PSGL-1 peptide: GST-ATEYEYLDYDFL; fluorescent peptide: LDYGE(DAP-pyrene)A, DAP-pyrene is a synthetic amino acid, (_L_-2,3-diaminopropionic acid conjugated with a pyrene); ^c^ The data was calculated from V_max_ based on an average molecular weight (52,000) of the enzyme; ^d^ The data was calculated from V_max_ based on the molecular weight of *h*TPST1 (42,186), *h*TPST2 (41,909) and *dm*TPST (39,860) estimated from protein database of NCBI [127]; ^e^ The unit of these data was s^−1^ in the original article.

#### 4.2.1. The Catalytic Mechanism of TPST

The crystal structure of TPST-PAP and TPST-PAP-substrate complexes have been solved and published in the Protein Data Bank (PDB, PDB No.: 3AP1, 3AP3) [47]. This structure provides basic information for structure/function relationship studies. Little research regarding the mechanisms of TPST has been reported. However, comparison among the structures of TPST and cytosolic sulfotransferases reveals some of their similarities in biochemical properties. Structurally, both types of sulfotransferases comprise a single α/β fold with a four- or five-stranded parallel sheet surrounded by helices [45,128]. Both the amino acid sequence and the structure of PAPS binding site are conserved for TPST and cytosolic sulfotransferase [47]. Thus, it is expected that tightly bound PAP and its inhibitory property to TPST is similar to those of cytosolic sulfotransferases [36,129,130]. In general, TPST1 and TPST2 exhibit similar activity with some variations. The kinetic constants and catalytic mechanism of TPST2 were investigated by MS [36]. The results suggest a two-site Ping-Pong model for TPST2 action, which indicates that the enzyme allows independent binding of substrates to two distinct sites. This mechanism model involves the formation of a sulfated enzyme covalent intermediate (sulfohistidine), but the intermediate has not been found [36]. Variations between TPST1 and TPST2 have been observed [56]. Human TPST displayed acidic pH optima at pH 6.5 and 6.0 for TPST1 and TPST2, respectively. They also varied in Mn^2+^ stimulatory effect. As shown in Table 4, K_m_ and V_max_ can be significantly different between those of TPST1 and TPST2 implying their differential substrate specificity and may have complementary functions in physiological conditions.

#### 4.2.2. The Role of Surrounding Amino Acids on Sulfated Tyrosine Residues

Analysis of the amino acids surrounding tyrosine residues subjected to PTS reveals the presence of acidic residues, glutamic and aspartic acids, within +5 to −5 positions of the tyrosine [131]. Replacement of acidic amino acids surrounding sulfation site decreased the activity of TPST [132]. It has also been proposed that the charge of the residue in the amino-terminal (−1) position of the tyrosine is critical and can be neutral or acidic, whereas a basic residue abolishes sulfation and increased the K_m_ value [133]. Structure and activity of human TPST2 indicates that the negative charge of glutamic acid (‒1 position) is recognized by backbone amide nitrogen, but not positively charged amino acid [47].

#### 4.2.3. The Significance of Multiple Tyrosine Sulfations

TPST substrates containing multiple tyrosine sulfation sites are common. These proteins include CCR5, CCR8, and PSGL-1 that have been found to be involved in important physiological and pathological mechanisms [10,89,134,135]. The question why more than one tyrosine is needed in the sulfation site of many TPST substrates is intriguing. Only two of the three potential sulfated tyrosines of human PSGL-1, Y46 and Y51, but not Y48, were found to be important for PSGL-1 binding to L-selectin [136]. The sulfation of Y48 and Y51 may promote virus binding and infection to host cells in EV71 [89]. In a previous study, as shown in Table 4, sulfated tyrosine significantly affects the next PTS by TPST [126]. The result indicates that TPST2 is more effective (10-fold) than TPST1 in catalytic efficiency (*k*_cat_/K_m_) using sY15CCR8 or CCR8 as a substrates. Effect of a monosulfated substrate (sY15CCR8) decreased its K_m_ value catalyzed by TPST1 and TPST2 (Table 4) by four- to five-fold. This indicates that sulfated tyrosine surrounding the sulfation site significantly affects the substrate recognition of TPST. At the current stage, although some noteworthy features on TPST and its substrates have been revealed, a majority of the detailed structure/function relationships and their reaction mechanisms remain to be elucidated.

## 5. Conclusions

The importance of post-translational PTS of membrane and secretory proteins is recognized in biology. However, much of the PTS remains unknown and only a small fraction of the biological functions of PST have been revealed. This review focuses on the biochemistry of PTS, which is fundamental for any further understanding of the role of PTS in biology. Although significant progress has been reported recently, basic questions concerning the biochemistry of PTS, such as the properties of TPST, its substrates, and the reaction mechanism(s), are largely unanswered.

TPST, the enzyme responsible for biological PTS, is now well known to exist in a variety of organisms. However, the majority of the TPST substrates may remain unknown. The TPST activities reported in the literature differ over a wide range, which may be due to the differences in preparation of the TPST or the reaction conditions. This implies that appropriate methods for the identification PTS or the activity of TPST are needed. It would be very useful to prepare antibodies that recognize various protein sulfation sites. Modern techniques have revealed the sequence and structure of TPST, which provide important information for the study of the mechanism of PTS and TPST action, however, few reports in the literature have been devoted to the study of the kinetics and chemical mechanism of TPST. The post-translational PTS is an open and attractive research area for chemists, biochemists, and biologists to explore for novel findings.
